# Successful surgical repair in an older adult with supracardiac total anomalous pulmonary venous connection: A case report

**DOI:** 10.3389/fcvm.2023.1121037

**Published:** 2023-03-22

**Authors:** Chunle Wang, Xia Xie, Huanwei Zhuang, Zhonghua Huang, Chukwuemeka Daniel Iroegbu, Mi Tang, Chengming Fan, Jinfu Yang

**Affiliations:** Department of Cardiovascular Surgery, The Second Xiangya Hospital, Central South University, Changsha, China

**Keywords:** TAPVC, older adult, rare, congenital heart disease, surgery

## Abstract

Total anomalous pulmonary venous connection (TAPVC) is a rare, cyanotic and critical congenital heart disease where the entire left and right pulmonary veins fail to drain into the left atrium directly. Also, TAPVC-induced tissue hypoxia gradually worsens after birth. Thus, timely surgical repairs are recommended once diagnosed, particularly with pulmonary venous drainage obstruction(s). Nonetheless, in sporadic cases, patients with TAPVC survive to adulthood with no surgical treatment. Herein, we report a 46-year-old female with TAPVC, where the four pulmonary veins drain into to the innominate vein (IV) *via* the vertical vein. The patient developed palpitations and non-anginal chest pain following routine activities for over three months. The patient had a successful surgical correction with excellent postoperative recovery.

## Background

Total anomalous pulmonary venous connection (TAPVC), predominantly diagnosed at newborn periods, is a critical congenital heart disease (CHD) where the entire four pulmonary veins fail to drain into the left atrium (LA) directly (1). TAPVC may occur as isolated lesions or in combination with other cardiac malformations including atrial septal defect (ASD) and ventricular septal defect (VSD). Notably, neonates with obstructed TAPVC may become critically ill shortly after birth. Without surgical repair, 75% to 80% of patients die by age one (2). Previously, where severe pulmonary vein obstruction was absent after performing atrial septostomy with balloon, elective surgical repair would be scheduled by two years of age or once diagnosed (3). However, such methods in most surgical institutions worldwide, including China, have become absolute. Instead, early anatomical repair, even in a pre-term neonate, is a widely accepted strategy. Hence, adult patients with TAPVC are seldom seen.

Supracardiac TAPVC is the best known type of abnormal pulmonary venous connection. In supracardiac type of TAPVC, the entire left and right pulmonary veins merges into a common pulmonary vein, drains through a so called “vertical vein”, then to the superior vena cava (SVC) and finally to the right atrium (RA). Patients with type of TAPVC had almost normal oxygen saturation which demonstrated almost no cyanosis. However, this type of TAPVC demonstrated the high pulmonary flow like atrial septal defect with large Left-to-right shunt. TAPVC has been described that one in 7,809 neonates born in America every year is diagnosed with TAPVC (4). Interestingly, in an extensive publication series on congenital cardiac diseases, supracardiac TAPVC was prevalent (47%), with the IV as the most common connection site (36% of all cases) (5).

Patient with TAPVC survival into adulthood is <7%, and often seen in the supracardiac type without the obvious pulmonary veins obstruction (6). Therefore, it was very rare for a 46-year-old female who referred to the cardiac center with palpitations and non-anginal chest pain after routine activity over three months and finally turned out to be a patient with TAPVC.

## Case presentation

A small-statured 46-year-old female weighing 34 kg was admitted to our department following palpitations and non-anginal chest pain after routine activities lasting over three months. The patient admitted successful pregnancy history while denying issues such as hypertension, diabetes, other cardiac health comorbidities, or psychosocial cases. She also denied notable medical, surgical history and cardiovascular family history. Also, physical examination showed weak body development. The patient's blood pressure was 108/78 mmHg. In addition, the extremity pulse oxygen saturation from both fingers and toes was 93%–96% without oxygen inhalation.

**Figure 1:**
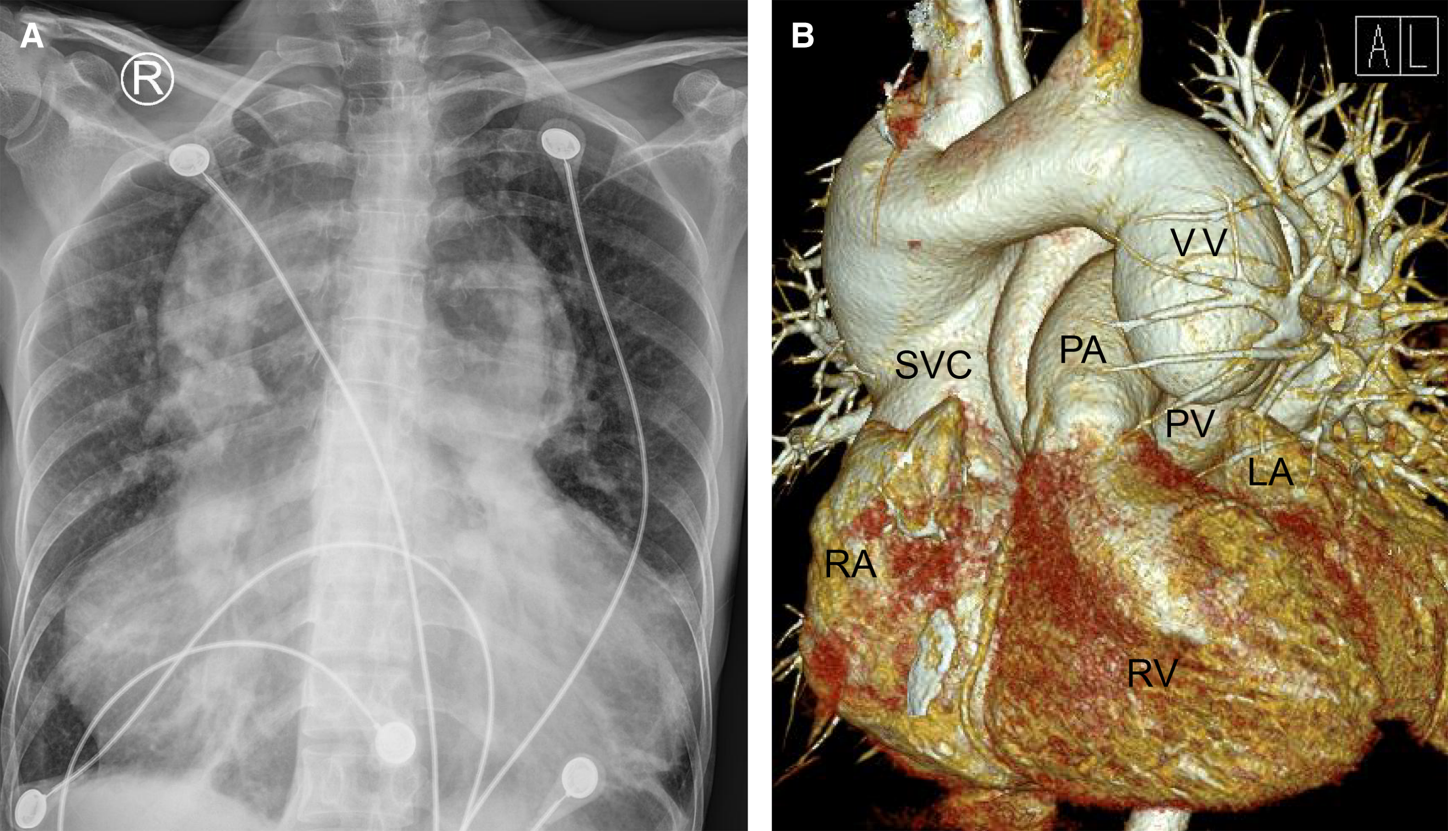
Chest radiography (**A**) and cardiac computered tomograhy angiography (**B**) preoperatively indicated that the lungs were clearly textured, the heart shadow was significantly enlarged (**A**); the left and right pulmonary veins were not connected to the left atrium but merged into a vertical vein and drained upward into the left brachiocephalic vein, innominate vein and then the superior vena cava. (**B**) LA, left atrium; PA, pulmonary artery; PV, pumonary vein; RA, right atrium; RV, right ventricle; SVC, superior vena cava; VV, vertical vein.

Auscultation revealed a heart rate of 106 bpm, heart sound enhancement, P2 hyperactivity, a right ventriclar outflow tract murmur and a tricuspid diastolic murmur. Physical palpation examination suggested a fluctuated heart apex with a palpable tremor. The routine laboratory examination results were typical except for a high BNP level (1737 pg/ml). Furthermore, the ECG indicated a significant atrial flutter and a right bundle branch block (Supplementary Figure S1A). Preoperative chest radiography indicated that the lungs were textured with a widened mediastinum and a significantly enlarged cardiothoracic ratio of 0.91 (Figure 1A).

**Figure 2:**
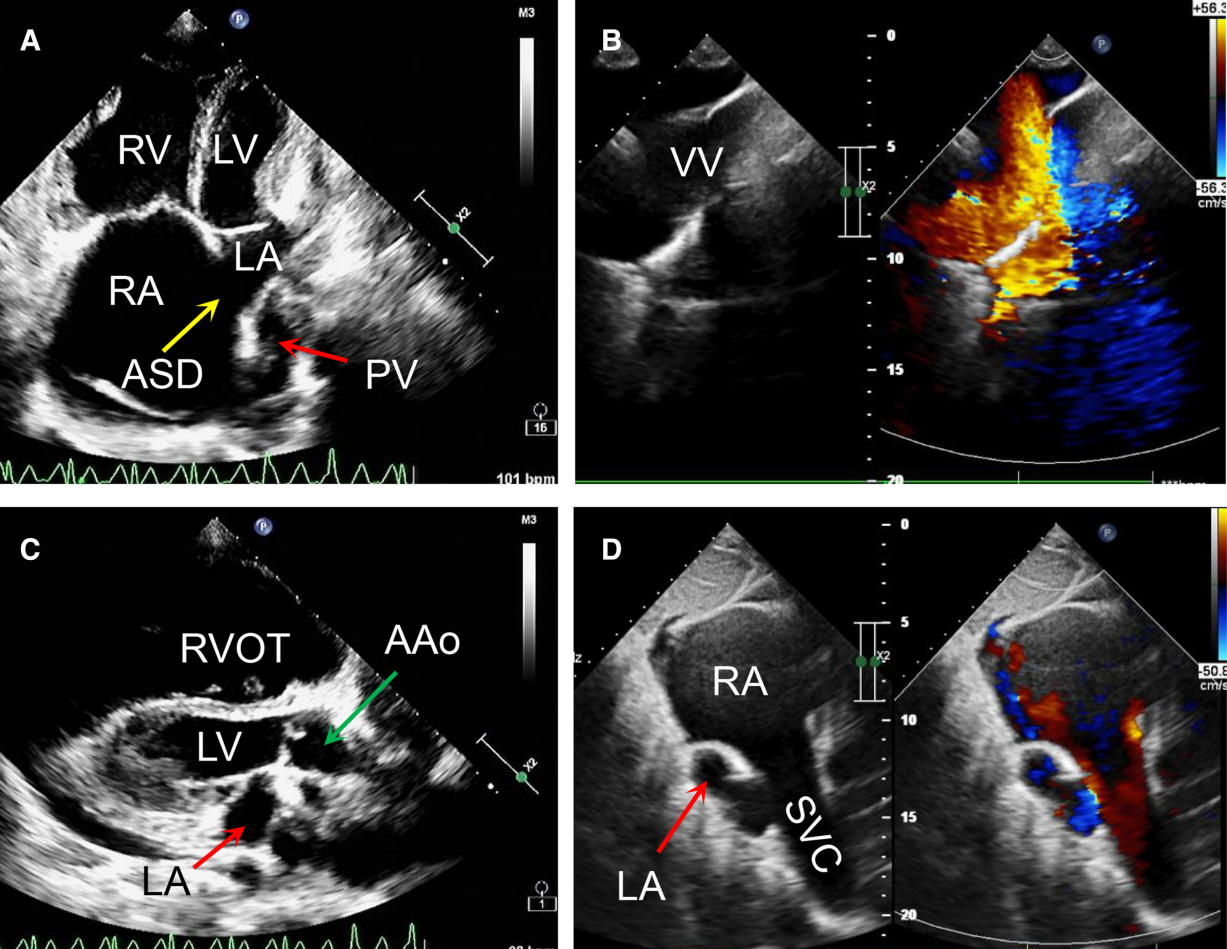
Transthoracic echocardiography preoperatively revealed that a right to left atrial septal shunt was detected with the diameter of around 40 mm (**A,B**); the ratio of ascending aorta to the pulmonary trunk was 24:32 (**C**); the left and right pulmonary veins returned to a vertical vein then innominate vein and the superior vena cava. (**D**) AAo, ascending aorta; ASD, atrial septal defect; LA, left atrium; LV, left ventricle; PV, pumonary vein; RA, right atrium; RV, right ventricle; RVOT, right ventriclar outflowtract; SVC, superior vena cava; VV, vertical vein.

On the other hand, a cardiac computer tomography angiography was performed. The three-dimensional reconstruction figure showed that the entire left and right pulmonary veins were not directly drained into the LA but merged into a vertical vein (with a diameter of 44 mm) and flowed upward into the left brachiocephalic vein, IV and then into the SVC (Figure 1B, Supplementary Video S1). Transthoracic echocardiography revealed an atrial septal defect at the lower part of the interatrial septum with right to left shunt and ∼40 mm in diameter (Figures 2A,B). The RA and right ventricle (RV) were enlarged with right ventricular outflow tract hypertrophy. The pulmonary movement was significantly widened, the mean pulmonary arterial pressure was 55 mmHg, and the pulmonary peak velocity was 1 m/s with a pressure gradient and acceleration time of 4mmHg and 50 ms, respectively. The diameter of ascending aorta and the pulmonary trunk were 24 mm and 32 mm, respectively (Figure 2C). The entire four pulmonary veins returned to a vertical vein (around 23 mm), the IV, and the SVC (Figure 2D). Moderate tricuspid regurgitation was also detected. The LA, LV, RA, and RV sizes were 29 mm, 27 mm, 59 mm, and 51 mm, respectively. The LVEDV and LVESV were 28 ml and 10 ml respectively. The EF was 64%, while CO was 2.2 L/min.

Cardiac catheterization and pulmonary angiography were further performed. They showed that the catheter entered the pulmonary vein from the SVC, significantly increasing the oxygen content, further confirming the supracardiac TAPVC diagnosis (Supplementary Video S2). The systolic and diastolic pulmonary pressure was 28 mmHg and 9 mmHg, respectively, with the mean at 18 mmHg. The pulmonary vascular resistance was 0.87 Wood unit and the Qp/Qs is 7.09.

**Figure 3:**
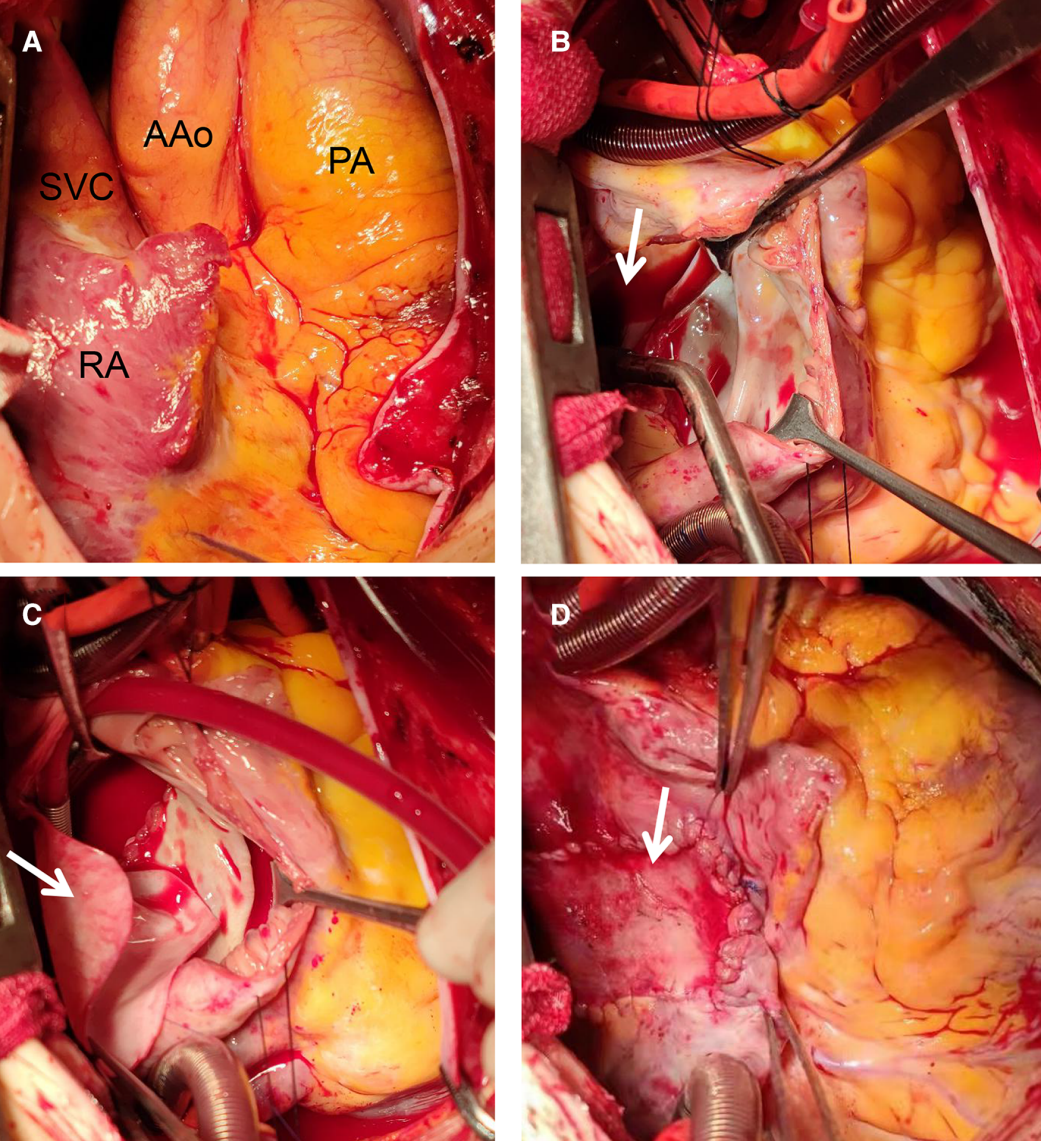
Intraoperative view of repair procedure: during the operation, the right atrium, right ventricle, pulmonary artery and superior vena cava were found significantly enlarged (**A**); A double-chamber incision was made and the posterior wall of the left atrium was opened (**B**, arrow); the common pulmonary veins were anastomosed with the posterior wall of the left atrium. A proper size of the bovine pericardium patch (**C**, arrow) was used to close the ASD and enlarged the left atrium. A proper size of autologous pericardium was used to close the right atrial incision (**D**, arrow). AAo, ascending aorta; PA, pumonary artery; RA, right atrium; SVC, superior vena cava.

At this juncture, the TAPVC diagnosis was certain, with no contraindication to surgery. Hence, a supracardiac TAPVC surgical repair was scheduled and performed (Figure 3). The procedure was performed under CPB, moderate hypothermia (28–30 °C) and *via* a median sternotomy. The surgical procedure involves transecting and permanent ligating the vertical vein, creating an anastomotic stoma between the LA and the venous fusion, and ASD closing. During the operation, it was detected that the RA and RV were enlarged. Also, the pulmonary artery, IV, and SVC were significantly enlarged. The diameter ratio of the ascending aorta to the pulmonary trunk was 1 : 2.5 (Figure 3A). The entire four pulmonary veins form a confluence above the LA, passing through the vertical vein, the IV and into the SVC. The atrial defect was located at the secondary orifice (∼40 mm in diameter) with moderate tricuspid valve regurgitation. A double-chamber incision was made including the incision from the LA posterior wall (Figure 3B, arrow). The incision was ∼4 cm along the long axis of the common pulmonary veins. The common pulmonary veins were anastomosed with incision from the the posterior wall of the LA. The bovine pericardium patch was adequately sized to close the ASD and enlarge the LA (Figure 3C). A 28 mm band (SOVERING TRICUSPID BAND^TM^, Sorin Group Italia Srl, Italy) was selected for the tricuspid repair. The autologous pericardium was adequately sized to close the right atrial incision (Figure 3D).

**Figure 4:**
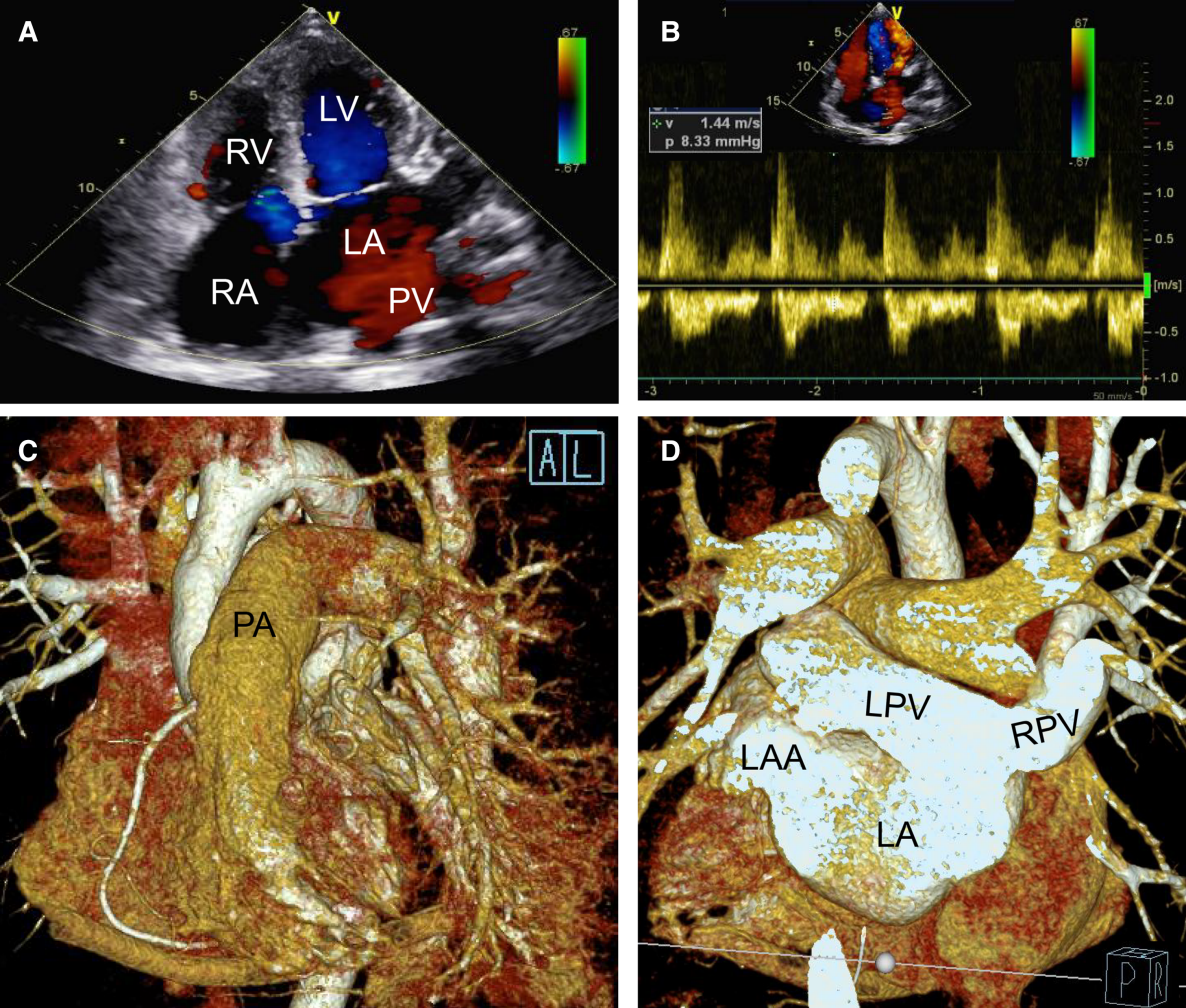
Postoperative transthoracic echocardiography (**A,B**) and cardiac CTA with three dimensional reconstruction figure (**C,D**) showing that the deformity correction was satisfactory without residual shunt (**A**) and pulmonary veinous obstruction (**B**) but with left and right pulmonary veins connected to the left atrium directly (**C,D**). LA, left atrium; LAA, left atrium; LPV, left pulmonary vein; LV, left atrial appendage; PA, pumonary artery; PV, pumonary vein; RA, right atrium; RPV, right pulmonary vein; RV, right ventricle.

During the operation and after completely closing the ASD (II), we detected no pulmonary vein obstruction. Likewise, the left ventricle functioned well without mitral or aortic regurgitation (as seen on transesophageal echocardiography). Postoperative transthoracic echocardiography (Figures 4A,B), cardiac CTA, and a three-dimensional reconstructed figure (Figures 4C,D) showed that the surgical correction was satisfactory with entire pulmonary veins directly connected to the LA. The time consumption of CPB, aortic cross-clamp, mechanical ventilation, and post-surgery ICU stay were 139 min, 81 min, 4 days, and 30 days, respectively. The patient recovered well and was discharged without complications. The ECG at discharge indicated a significant atrial flutter and a right bundle branch block same as pre-operatively (Supplementary Figure S1B). There were no postoperative oral drugs used to eliminate pulmonary vascular resistance. Transthoracic echocardiography half-year, 1-year and 2-years post-surgery showed that the deformity correction was satisfactory without any anastomotic obstruction or residual shunt.

## Discussion and conclusions

TAPVC is a critical CHD accounting for <1% of all CHDs (7). TAPVC is characterized by the absence of direct connection between the pulmonary venous and the LA. TAPVC includes four subtypes accprding to where the anomalous veins drain: supracardiac, cardiac, infracardiac, and mixed (8). Supracardiac type of TAPVC is the best known type of abnormal pulmonary venous connection, accounting for 47% of all, with the mixed pattern being the rarest type. Notably, TAPVC coronary type, although it belongs to the intracardiac type, refers to the confluent vessel horizontally connecting to the RA *via* the coronary sinus and accounts for 20%–30% of the general (9). However, depending on its course, the anomalous vein may divide into two types: obstructed and non-obstructed. Nonetheless, an obstructed pulmonary venous connection represents a life-threatening neonatal emergency requiring urgent surgery.

Since its first attempt to correct the abnormal pulmonary venous connection in 1951 (10), numerous advanced surgical techniques have significantly emerged and improved the outcomes of TAPVC patients. Notwithstanding, TAPVC surgical correction remains a challenge, with a 10% to 20% early mortality rate (11–13). In TAPVR without pulmonary veins obstruction, high pulmonary vascular resistance (Eisenmenger Syndrome) develops late, nonetheless, with the patient's age, it is not a non-essential fact (the pulmonary vascular resistance was 0.87 Wood). The patient exhibited symptoms including palpitation, fatigue and dyspnea after routine activity; hence, she required a radical surgical repair.

Thus, if corrective surgery were not performed, the patient would develop moderate and then severe pulmonary hypertension causing severe tissue hypoxia, heart failure, and death. Surgical radical operation aims at creating a smooth and unobstructed drainage from the entire pulmonary veins to the LA. Therefore, a postoperative anastomotic stoma obstruction with pulmonary venous hypertension is a severe complication and may increase morbidity and mortality (14–16).

The single-patch technique uses only one patch to drain pulmonary vein blood from the SVC to the LA. Rhythm disturbance and SVC obstruction are known procedural complications due to an inadequate blood supply to the sinus node during surgery (17). Schuster and colleagues used an extra patch (two-patch technique) to broadening the SVC–RA junction to achieve an unobstructed SVC drainage and, thus, reproducible without an increase in complications, such as pulmonary venous obstruction or SVC stenosis or any rhythmic issues (18).

Most frequently, the two-patch technique was used for the repair of extra-cardiac type of TAPVC. A longitudinal incision is performed along the pulmonary venous confluence, then the right side of the anastomosis between the LA and the pulmonary venous sinus is broadened *via* the first patch. Simultaneous LA enlargement is achieved by a rightward shifting of the second patch used for ASD closing. Then the first patch is flipped to complete the RA enlargement (19). As described by A Corno (20), a bi-atrial transverse incision was performed. The technique enhances the pulmonary veins exposure (a significant advantage) while increasing the LA size with the two-patch technique and reducing postoperative obstruction occurrences. Nonetheless, the method has more suture lines on the LA and RA, raising the atrial arrhythmias' possibility incidence rate compared to the others (19).

The oldest reported patients with a successful TAPVC repair or without any TAPVC repair were aged 57 years and 62 years old, respectively (21, 22). Although TAPVC is rarely discovered in adulthood, pregnancy and delivery could be achieved if the pulmonary venous drainage resistance is not significantly increased (23). In 1994, a patient with TAPVC was reported to have a successful pregnancy at her 29 years of age (24).

The presented patient has survived into adulthood and has a successful pregnancy and delivery history, primarily because she had a sizeable non-restrictive ASD without obvious pulmonary venous drainage obstruction (25). All her pre-operation data indicated that the pulmonary vascular resistance was not high, and an ASD complete closure could be considered. However, if CPB withdrawal during the operation were unsuccessful, we would opt for the “unidirectional flap patch” technique.

The case herein shows that the unobstructed pulmonary venous drainage and adequate right-to-left shunt may maintain a satisfactory cardiac output and prevent pulmonary venous obstruction. Finally, late diagnosis may occur in adult TAPVC patients, but a surgical repair may still be possible (26).

## Data Availability

The raw data supporting the conclusions of this article will be made available by the authors, without undue reservation.
