# Supplementary figures and images for: Successful surgical repair in an older adult with supracardiac total anomalous pulmonary venous connection: A case report
Source: Front Cardiovasc Med. 2023 Mar 22;10:1121037. doi: 10.3389/fcvm.2023.1121037 (PMC10073726; doi:10.3389/fcvm.2023.1121037)

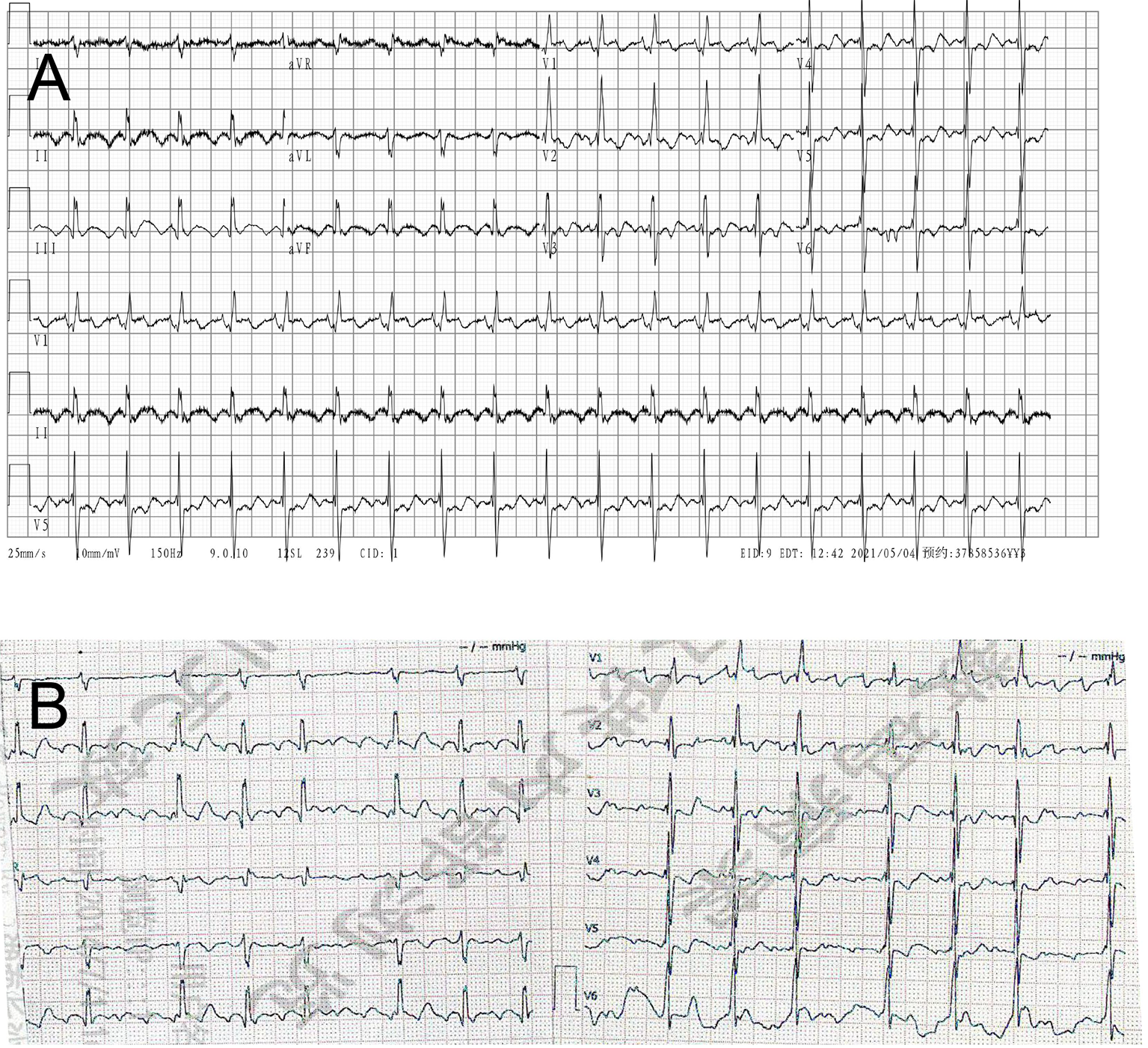

Supplement: Supplementary file 1 [file Image1.tif]
