# Supplementary material for: Successful surgical repair in an older adult with supracardiac total anomalous pulmonary venous connection: A case report
Source: Front Cardiovasc Med. 2023 Mar 22;10:1121037. doi: 10.3389/fcvm.2023.1121037 (PMC10073726; doi:10.3389/fcvm.2023.1121037)
Supplement: Supplementary file 4 [file Table1.docx]

**Supplemental Table 1.** Characteristics of the Patient

| Age (m) | Body Weight (kg) | Gender | Pr-eoperative blood pressure (mmHg) | Pre-operative extremity pulse oxygen saturation (%) | Pre-operative heart rate (bpm) | Pre-operative BNP level ( pg/ml ) | Pre-operative mean pulmonary arterial pressure (mmHg) | Pulmonary vascular resistance Pre-operatively (wood) | The size of LA, LV, RA and RV Pre-operatively (mm) |
| --- | --- | --- | --- | --- | --- | --- | --- | --- | --- |
| 46 | 34 | F | 108/78 | 93-96 | 106 | 1737 | 55 | 0.87 | 29, 27, 59 and 51 |
| CPB time (min) | X-aorta time (min) | Mechanical ventilation time (hour) | Blood pressure at discharge (mmHg) | Extremity pulse oxygen saturation at discharge (%) | Heart rate at discharge (bpm) | BNP level at discharge ( pg/ml ) | Mean pulmonary arterial pressure at discharge (mmHg) | ICU LOS (day) | The size of LA, LV, RA and RV at discharge (mm) |
| 139 | 81 | 49 | 105/68 | 99 | 87 | 353 | 28 | 4 | 29, 37, 33 and 34 |

**Table 2.** Operative Details and Outcomes of Patients Undergoing Sliding Arch Aortoplasty for Hypoplasia and COA

| Patient Number |  | | Variables | | | | | | | | |
| --- | --- | --- | --- | --- | --- | --- | --- | --- | --- | --- | --- |
|  | CPB time (min) | X-aorta time (min) | | SCP time (min) | Posoperative Right Arm-Left  Leg gradient (mmHg) | Peak velocity on Echo(m/s) | Surgical procedure | Concomitant procedures | Mechanical ventilation time (day) | ICU LOS (day) | Hospital LOS (day) |
| 1 | 137 | 86 | | 15 | 0 | 1.2 | Technique 1 | VSD repair | 1 | 2 | 11 |
| 2 | 176 | 91 | | 22 | 0 | 1.2 | Technique 2 | VSD,ASD,PDA repair | 2 | 3 | 11 |
| 3 | 157 | 72 | | 25 | 0 | 1.7 | Technique 1 | VSD repair | 2 | 3 | 12 |
| 4 | 186 | 100 | | 24 | 5 | 1.5 | Technique 1 | VSD,ASD,PDA repair | 1 | 2 | 14 |
| 5 | 133 | 71 | | 21 | 8 | 2.0 | Technique 1 | VSD repair | 3 | 5 | 15 |
| 6 | 159 | 83 | | 17 | 0 | 1.7 | Technique 2 | VSD,ASD repair | 1 | 2 | 12 |
| 7 | 166 | 87 | | 24 | 5 | 1.2 | Technique 2 | VSD,ASD,PDA repair | 1 | 2 | 13 |
| 8 | 161 | 79 | | 23 | 4 | 1.3 | Technique 1 | VSD,ASD repair | 2 | 4 | 15 |
| 9 | 132 | 58 | | 19 | 7 | 1.4 | Technique 1 | VSD repair | 1 | 2 | 11 |
| 10 | 141 | 77 | | 32 | 0 | 1.2 | Technique 2 | VSD,ASD,PDA repair | 3 | 5 | 18 |
| 11 | 148 | 65 | | 28 | 0 | 1.6 | Technique 1 | VSD repair | 4 | 7 | 22 |
| 12 | 161 | 68 | | 21 | 2 | 1.5 | Technique 2 | VSD,ASD repair | 1 | 3 | 15 |
| 13 | 180 | 90 | | 30 | 0 | 1.2 | Technique 2 | VSD,ASD,PDA repair | 1 | 2 | 12 |
| Mean±SD | 160±15 | 80±31 | | 23±10 | 2.4±3.0 | 1.4±0.3 |  |  | 1.8±1.0 | 3.2±1.6 | 13.9±3.2 |
| Median (range) | 159(132~186) | 79(58~100) | | 23(15~32) | 0(0~8) | 1.4(1.2~2) |  |  | 1(1~4) | 3(2~7) | 13(11~22) |

CPB: cardiopulmonary bypass; X-aorta: aortic cross-clamp SCP: selective cerebral perfusion; VSD: ventricle septal defect, ASD: atrial septal defect, PDA: patent ductus arteriosus; LOS:longth of stay
